# Overexpression of zinc-finger protein 677 inhibits proliferation and invasion by and induces apoptosis in clear cell renal cell carcinoma

**DOI:** 10.1080/21655979.2022.2038891

**Published:** 2022-02-14

**Authors:** W Liang, Sh Chen, Gl Yang, Jy Feng, Q Ling, B Wu, Hb Yan, Jw Cheng

**Affiliations:** Department of Urology, The First Affiliated Hospital of Guangxi Medical University, Nanning, People’s Republic of China

**Keywords:** ZNF677, clear cell renal cell carcinoma, dna methylation, emt

## Abstract

Recent studies have demonstrated that zinc-finger protein 677 (ZNF677) acts as a tumor suppressor gene in cancer. However, the expression and function of ZNF677 in clear cell renal cell carcinoma (ccRCC) are still unclear. In this study, we used bioinformatics analysis and in vitro experiments to investigate the expression of ZNF677 in ccRCC tissues and the malignant biological behavior of ZNF677 in 786–0 cells. We demonstrated that ZNF677 is hypermethylated in ccRCC and is associated with clinicopathological features. The results of the functional assays indicate that ZNF677 inhibits tumor cell proliferation and invasion and induces apoptosis. Further prognostic analysis indicated that low expression of ZNF677 is associated with shorter overall survival. Additionally, ZNF677 overexpression suppressed the invasion and epithelial-mesenchymal transition of 786–0 cells by inactivating the PI3K/AKT signaling pathway. This is the first report to evaluate the influence of ZNF677 on ccRCC cells malignant biological behavior. The results indicate that high expression of ZNF677 could be considered as a favorable prognostic indicator for ccRCC.

## Graphical Abstract

**Figure:**
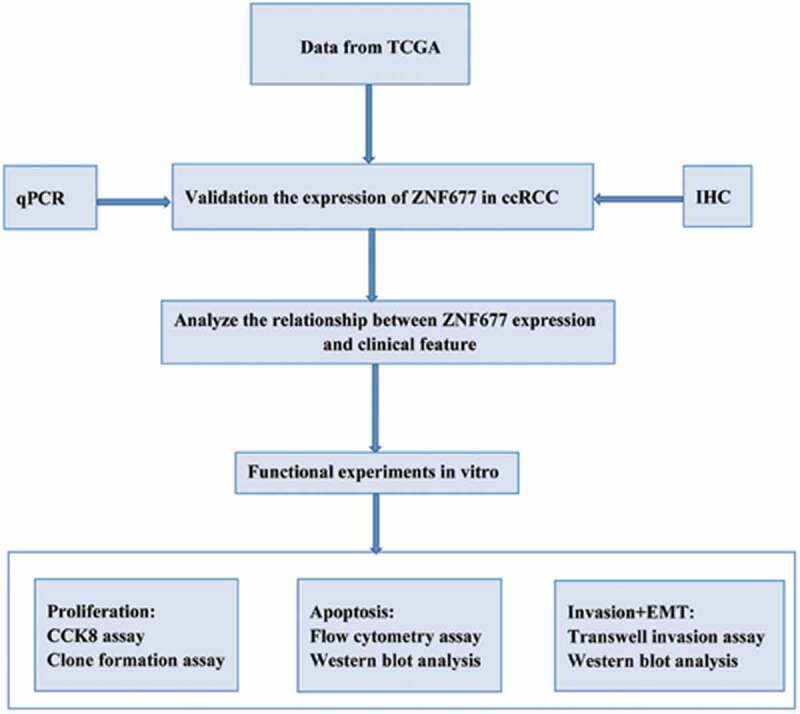

## Introduction

Renal cell carcinoma (RCC) is the third most prevalent urological malignancy worldwide, ranking sixth and eighth among all cancers in men and women, respectively [1]. The morbidity of RCC is rising by 2–4% each year [1]. The main subtype of RCC is clear cell renal cell carcinoma (ccRCC), which accounts for about 70–80% of all RCC cases. The most effective therapeutic strategy is partial or radical nephrectomy for localized ccRCC, depending on the tumor size and location, and the 5-year survival rate is approximately 93% [1]. Unfortunately, for some patients, ccRCC has already metastasized by the time they are initially diagnosed, which dramatically reduces the 5-year survival rate [2]. Advanced ccRCC is not sensitive to radiotherapy or chemotherapy. At present, targeted therapy is an important therapeutic method for ccRCC [2]. However, the prognosis of ccRCC is still unsatisfactory due to drug resistance. Therefore, it is imperative to identify novel biomarkers and therapeutic targets that may improve therapeutic effects on ccRCC.

The occurrence and development of cancer are closely related to the activation of oncogenes or inactivation of tumor suppressor genes by epigenetic modifications, including DNA methylation, chromosome remodeling, histone modification, and non-coding RNA regulation [3]. In recent years, many molecular biomarkers of clear cell renal cell carcinoma (ccRCC) have been explored. For example, ABCG1 and P4HB may serve as independent diagnostic and prognostic markers for ccRCC [4,5]. CYP2J2 has been shown to have a higher expression in ccRCC and prolong the survival rate of ccRCC patients [6]. Transcription factors play an important role in the regulation of gene expression. Zinc-finger proteins (ZNFs), which have a ‘finger-like’ domain, constitute the largest transcription factor family in the human genome. Most ZNFs belong to the Krüppel-associated box domain ZNF (KRAB-ZNF) superfamily. These ZNFs bind to DNA, RNA, and proteins through their specific zinc finger (ZF) structure and play an important role in cellular differentiation, proliferation, apoptosis, invasion, and metastasis [7–9]. Over the last few decades, a substantial body of literature has revealed that ZNFs play a crucial role in the occurrence and development of cancer. For instance, high expression of ZNF143 has been correlated with lymph node metastasis in gastric cancer, indicating that ZNF143 plays an important role in gastric cancer metastasis [10]. ZNF281 can activate the Wnt/β-catenin signaling pathway and enhance the invasion and proliferation of pancreatic cancer cells [11]. ZNF830 acts as an oncogene in patients with lung and gastric cancers and is correlated with poor outcomes [12].

ZNF677, which comprises C2H2-type ZFs and a KRAB domain and is a member of the KRAB-ZFP family, is located in the chromosomal region 19q13, where a frequent loss of heterozygosity occurs in cancer [13]. Sequence analysis demonstrated that the ZNF677 promoter contains a CpG Island, implying that CpG methylation may be involved in silencing its expression in cancer. Recent studies have demonstrated that ZNF677 is expressed at low levels and functions as a tumor suppressor in thyroid, lung, and gastric cancers as a result of DNA methylation [13–15].

A recent study reported that ZNF677 is aberrantly methylated in ccRCC and could serve as a noninvasive urine-based diagnostic and follow-up marker for ccRCC [16]. However, the function and mechanisms of action of ZNF677 in ccRCC cells remain elusive due to the lack of research in that area. Because of the significance of ZNF677 in cancer development, we hypothesized that it may play an important role in ccRCC tumorigenesis and metastasis. In this study, we aimed to determine the expression and function of ZNF677 in ccRCC.Our study was the first to investigate the influence of ZNF677 on ccRCC cells malignant biological behavior.

## Materials and methods

### Patients and samples

We included 60 ccRCC tissues and 47 normal renal tissues from patients with ccRCC at the First Affiliated Hospital of Guangxi Medical University, China, between 2015 and 2021. All histopathological parameters were evaluated according to 8^th^ tumor node metastasis classification (TNM) classification of the International Union Against Cancer (UICC). When analyzing the relationship between clinicopathological features and ZNF677 expression, we separated T as T1 and T2-T4 because of the different treatment methods. According to treatment guidelines, we usually perform partial nephrectomy for the T1 cases. For the T2-T4 cases, we usually perform radical nephrectomy.For the Fuhrman nuclear grading, it based on the degree of nuclear cell differentiation. I–II was always considered as high differentiation and III–IV was always considered as low differentiation.All samples were confirmed by histopathological examination and handled according to the ethical and legal standards. Ethical approval was obtained from the Ethics Review Committee of the First Hospital of Guangxi Medical University. Informed consent was obtained from all the enrolled patients.

### Cell culture and plasmid transfection

Cell culture and plasmid transfection was performed as previously described [17].The renal cancer cell line 786–0 and immortalized human proximal tubular cell line HK-2 were obtained from the Cell Bank of the Chinese Academy of Sciences (Shanghai, China). The cells were grown in a humidified CO_2_ incubator at 37°C and maintained in RPMI 1640 medium supplemented with 10% fetal bovine serum (FBS) and 1% penicillin-streptomycin (Invitrogen, Carlsbad, CA, USA). Both the negative control and plasmid DNA encoding human ZNF677 were purchased from Origene (Rockville, MD, USA) and subcloned into the pcDNA 3.1 plasmid vector according to the manufacturer’s instructions (Invitrogen). Transfection efficacy was confirmed by Western blotting.

### RNA extraction and quantitative real-time polymerase chain reaction (qRT-PCR)

RNA extraction and quantitative real-time polymerase chain reaction was performed as previously described [18]. RNA was extracted from ccRCC samples and 786–0 cells using the TRIzol RNA Isolation Reagent (Thermo Fisher Scientific) according to the manufacturer’s protocol. The first cDNA strand for ZNF677 was synthesized from 1 µg of RNA using the cDNA Reverse Transcription Kit (Thermo Fisher Scientific). The quantitative real-time polymerase chain reaction (qRT-PCR) was carried out using the SYBR® Premix Dimer Eraser Kit (TaKaRa, Dalian, China) on an Applied Biosystems 7,500 Real-Time PCR System. GAPDH was used as an internal control to normalize the expression of ZNF677. The 2− ΔΔCT method was used to calculate the relative expression of ZNF677. The primers used in this study were as follows: for GAPDH, forward, 5’-AAGGCCGGTTATCAACGT-3’; reverse, 5’-GCCAGTCCCTCACTGCTCT-3’; for ZNF677, forward, 5’-ACAAGCAAGGGATTATCACCAAA-3’; reverse, 5-CAGGCTGTCAAACTTAGGCAT-3.

### Western blot analysis

Western blotting was performed as previously described [18]. Protein extracts were separated by sodium dodecyl sulfate-polyacrylamide gel electrophoresis and transferred onto polyvinylidene difluoride membranes (Millipore, Bedford, MA, USA). The blots were blocked using 5% skim milk in Tris-buffered saline and Tween 20 (TBST) and then incubated with antibodies against glyceraldehyde 3-phosphate dehydrogenase (GAPDH) (ab16651, 1:150, Abcam, UK), ZNF677 (ab155075, 1:100, Abcam), caspase-3 (9662, 1:500, CST, Danvers, MA, USA), caspase-9 (9508, 1:500, CST), poly (ADP-ribose) polymerase (PARP) (9532, 1:300, CST), BCL-2 (ab32124, 1:100, Abcam), E-cadherin (3195, 1:500, CST), vimentin (5741S, 1:500, CST), and p-AKT (2965, 1:600, CST). After washing, the blots were incubated with horseradish peroxidase-conjugated secondary antibodies for 1 h at room temperature and visualized using a super-enhanced chemiluminescence chromogenic substrate (Applygen, Beijing, China). GAPDH was used as an internal control.

### CCK8 assay

A CCK8 assay was performed as previously described [18]. For the CCK8 assay, 786–0 cells were seeded in a 96-well cell culture plate (5 × 10^3^ cells/well) at 37°C for 24 h and then incubated with serum for another 6 h. Thereafter, the cells were transfected with pcDNA, ZNF677, or negative control pcDNA. On the day of cell growth rate assessment, 100 µL of spent medium was replaced with an equal volume of fresh medium containing 10% CCK8 and the cells were incubated at 37°C for 3 h, following which the absorbance was determined at 450 nm using a microplate reader.

### Clone formation assay

A clone formation assay was performed as previously described [18]. The 786–0 cells were seeded in 12-well plates (5 × 10^2^ cells/well) and incubated under standard conditions for 1–2 weeks. Phosphate-buffered saline (PBS) was used to wash the plates twice, and the plates were fixed with 4% paraformaldehyde for 10 min at room temperature. After staining with 0.1% crystal violet for 30 min, the cells were photographed, and the number of colonies was counted.

### Flow cytometry assay

A flow cytometry assay was performed as previously described [18]. After 48 h of transfection, the transfected cells in each well were collected and subjected to apoptosis detection using Annexin V-FITC/PI staining. Each sample was tested in triplicate, and analyses were performed using Kaluza Analysis 2.0 (Beckman Coulter, Inc.) according to the manufacturer’s guidelines.

### Transwell invasion assay

A transwell invasion assay was performed as previously described [18]. A 24-well transwell unit (Corning, New York, USA) with an 8 µm pore size polycarbonate filter was used following the manufacturer’s protocol. Briefly, 3 × 10^5^ cells were seeded into the upper chamber of the insert with Matrigel (BD, Franklin Lakes, NJ, USA) in serum-free medium. The lower chamber was filled with the medium and PBS. After incubation for 48 h, cells that invaded the extracellular matrix (ECM) were stained with methanol and crystal violet and counted in five random fields under a light microscope. The experiment was performed in triplicate.

### Tissue microarray (TMA) construction and immunohistochemical analysis

TMA construction was performed as Steurer S previously described [19]. For immunohistochemistry (IHC) examination, 60 archived formalin-fixed paraffin-embedded ccRCC tissues were collected and assembled into TMAs. The construction of TMA was performed as previously described. In addition, 47 samples from adjacent noncancerous renal tissues were also arrayed separately to serve as a reference normal control. A rotary microtome (Accu-Cut; Sakura, Torrance, CA, USA) was used to cut the TMA blocks into 0.4 mm thick paraffin sections, which were mounted onto special adhesive slides (SuperFrost Plus, Thermo Scientific). Monoclonal anti-ZNF677 antibody (ab155075, 1:100, Abcam) was used for IHC. Five random fields were selected on each slide for scoring, and the mean score of each slide was used in the final analyses. The percentage of positive tumor cells and the staining intensity were then multiplied to generate the immunoreactivity score (IS) for each tumor sample. Unequivocal nuclear and cytoplasmic staining patterns of ZNF677 were considered positive. The staining intensity was determined as follows: no staining, 0; weak staining, 1; intermediate staining, 2; and heavy staining, 3. The percentage of stained cells was scored as follows: 0–10% positive cells, 1; 10–50% positive cells, 2; and >50% positive cells, 3. The IHC scoring was performed by two independent pathologists without knowledge of the clinical features of the patients. Tissues from human pancreas were used as positive controls as recommended by the manufacturer. The negative controls were processed using normal rabbit serum (Dako, Carpinteria, CA, USA) as the primary antibody.

### UALCAN analysis

UALCAN (http://ualcan.path.uab.edu/) is an online, open-access platform that contains TCGA data, including gene expression, protein expression (CPTAC dataset), promoter methylation, miRNA expression, and clinicopathological data. In this study, UALCAN (http://ualcan.path.uab.edu/cgi-bin/ualcan-res.pl) was used to assess the expression and promoter methylation of ZNF677 in ccRCC as well as the prognosis of ccRCC using the TCGA database.

### Statistical analysis

All data analyses were performed using SPSS (version 22.0; SPSS Inc., Chicago, IL, USA). The χ^2^ test was used to analyze the differences in the expression pattern of RNA between the ccRCC and normal renal tissue groups and the relationship between ZNF677 expression and clinicopathological variables. Survival data were analyzed using Kaplan–Meier curves and log-rank tests. Statistical significance was set at *P* < 0.05.

## Results

In this study, we aimed to investigate the expression and function of ZNF677 in ccRCC. We hypothesized that ZNF677 acts as a tumor suppressor in the progression of ccRCC. Based on the results of bioinformatics analysis and in vitro experiments, we demonstrated that ZNF677 was significantly downregulated in ccRCC tissues and that its overexpression inhibited the proliferation and invasion, and promoted the apoptosis of 786–0 cells. Overexpression of ZNF677 suppressed the invasion and EMT of 786–0 cells through inactivation of the PI3K/AKT signaling pathway. Our study was the first to investigate the influence of ZNF677 on ccRCC cells malignant biological behavior.

### ZNF677 had a lower expression in ccRCC tissues and served as a tumor suppressor gene

**Figure 1.**
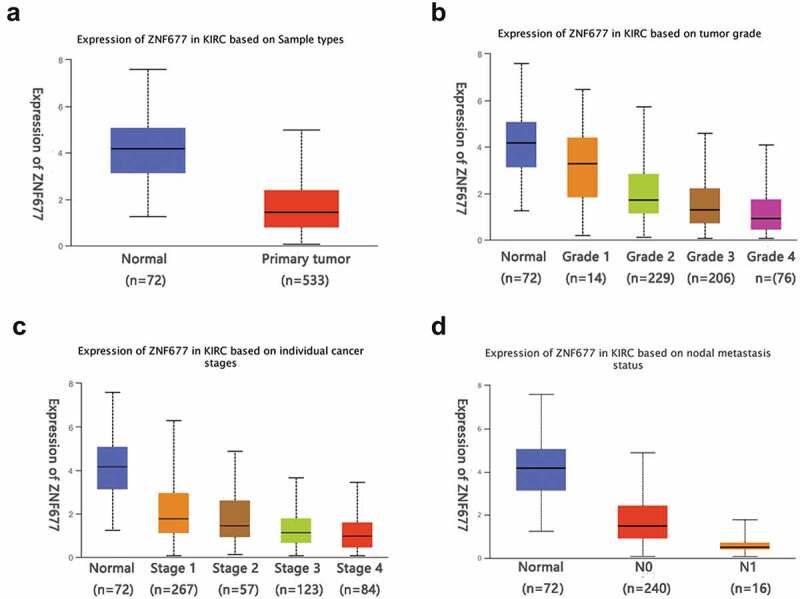
The online database showed that ZNF677 was considerably downregulated in tumor tissues. (a) Expression of ZNF677 in 533 ccRCC tissues and 72 normal tissues from the TCGA database. (b) Different expression of ZNF677 in ccRCC based on tumor grade. (c) Different expression of ZNF677 in ccRCC based on individual cancer stages. (d) Different expression of ZNF677 in ccRCC based on metastasis status.

**Figure 2.**
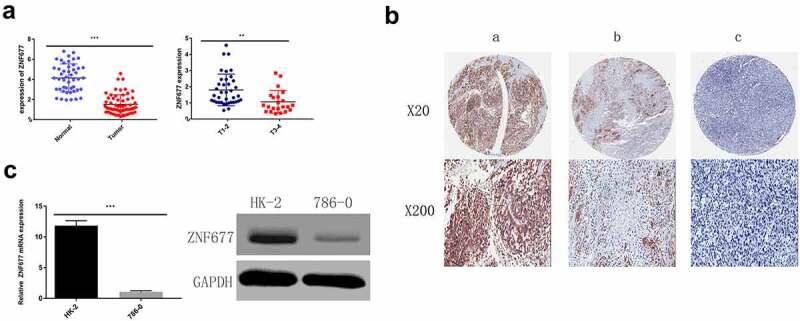
Expression of ZNF677 in ccRCC samples and normal tissues and 786–0 cells. (a) The relative expression levels of ZNF677 mRNA in 60 ccRCC tissues and 47 normal tissues were determined by qRT-PCR. (b) The relative expression levels of ZNF677 proteins in 60 ccRCC tissues and 47 normal tissues were measured by IHC. ZNF677 protein levels were found to be downregulated in cancer tissues in comparison to normal tissues. Moreover, the expression of the ZNF677 protein was gradually decreased in the primary tumor and metastatic tissues: (a) normal tissue, (b) non-metastatic cancer tissue, (c) metastatic cancer tissue. (C) The relative expression level of ZNF677 mRNA and protein in the ccRCC 786–0 cell line was clearly decreased compared with the immortalized human proximal tubular cell line HK-2 cells evaluated by qPCR and Western blot.

To evaluate the mRNA expression patterns of *ZNF677* in ccRCC, we first used the TCGA data of 533 ccRCC tissues and 72 normal tissues available from the UALCAN platform and found that *ZNF677* was considerably downregulated in tumor tissues compared to that in normal tissues (Figure 1a). The expression of *ZNF677* at different grades and stages of cancer malignancy is shown in Figure 1b–d. To validate the results of the TCGA data, the expression of ZNF677 mRNA and proteins in 60 tumor and 47 normal tissues was examined by qPCR and IHC, respectively. The qPCR results showed that *ZNF677* mRNA levels were downregulated in ccRCC tissues compared to that in normal tissues (Figure 2a). Furthermore, IHC analysis revealed that ZNF677 protein levels were downregulated in cancer tissues. Moreover, the expression of ZNF677 proteins gradually decreased from the primary tumor to metastatic tissues (Figure 2b). Staining was weak in the primary tumor tissues and absent in the metastatic tissues. The results of qPCR and Western blotting also showed that ZNF677 was downregulated in the 786–0 ccRCC cells than in immortalized human proximal tubular HK-2 cells (Figure 2c). These findings indicate that *ZNF677* may be a tumor suppressor gene in ccRCC, which is consistent with the findings of previous studies on gastric, lung, and thyroid cancer [13–15].

### Low expression of ZNF677 was due to hypermethylation and was correlated with clinicopathological features and poor prognosis

**Figure 3.**
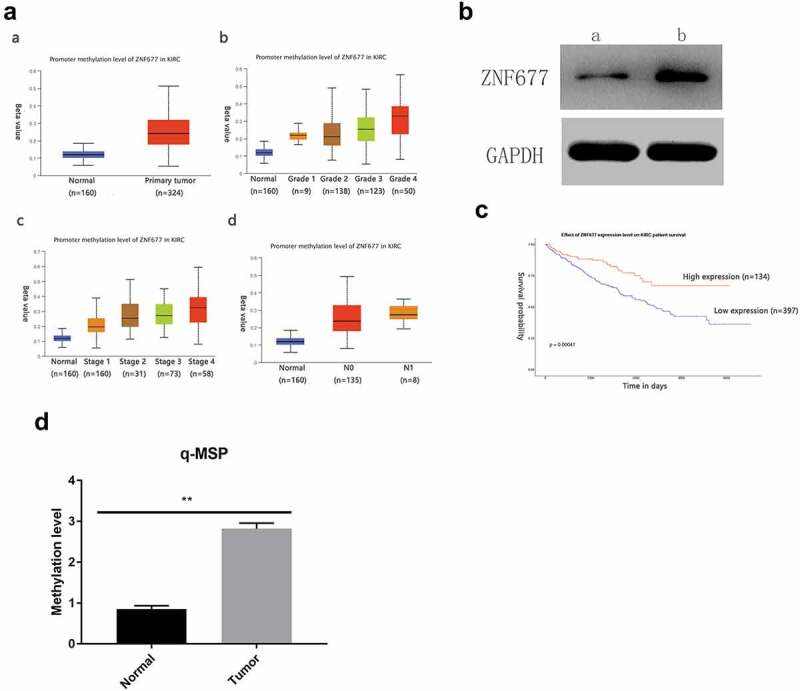
Low expression of ZNF677 was due to hypermethylation. (a) The methylation status of ZNF677 in primary ccRCC tissues and normal tissues from the TCGA database. (b) A drug demethylation experiment showed that treatment with the demethylation drug Aza combined with the HDAC inhibitor trichostatin A could restore the expression of ZNF677 in 786–0 cells, as assessed by Western blot: (a) drug (-), (b) drug (+). (c) The prognostic effect of ZNF677 expression on ccRCC from the TCGA database. Patients with higher ZNF677 mRNA expression levels had a longer survival time compared to those with low ZNF677 mRNA levels. (d) Q-MSP results showing ZNF677 methylation levels in ccRCC tissues and normal tissues.

ZNF677, which is often expressed in normal tissues, is decreased or absent in cancer tissues due to aberrant methylation. Therefore, we examined the methylation status of *ZNF677* in ccRCC. First, the methylation status of *ZNF677* in primary ccRCC tissues and normal tissues from the TCGA database was analyzed. As shown in Figure 3, ZNF677 methylation was more common in ccRCC tissues than in normal tissues (Figure 3a). We then evaluated *ZNF677* methylation in the collected ccRCC tissues using a methylation-specific assay. The results showed that *ZNF677* was methylated in 51 out of 60 (85%) ccRCC tissues and 2 out of 47 (4.2%) normal tissues (Figure 3d; Table 1), suggesting frequent methylation of ZNF677 in ccRCC. To further confirm whether ZNF677 expression is regulated by methylation, we treated 786–0 cells with the demethylation drug Aza combined with the histone deacetylase inhibitor trichostatin A (TSA). Western blot results indicated that the expression of ZNF677 was dramatically restored after treatment with Aza (Figure 3b). We then investigated the association between *ZNF677* methylation and the clinicopathological features of patients with ccRCC. The data showed that *ZNF677* methylation was significantly positively associated with pathological T stage, Fuhrman classification, and lymph node metastasis but not with age, sex, smoking, and distant metastasis (Table 2). To further explore the potential prognostic value of ZNF677 in ccRCC, a prognostic analysis was performed using the TCGA database. The results suggested that patients with higher *ZNF677* mRNA expression levels had a longer overall survival than those with low *ZNF677* mRNA levels (Figure 3c).

**Table 1.** Methylation status of ZNF677 between normal tissues and ccRCC tissues

ZNF677			
	Methylation	Unmethylation	Frequency of methylation
Normal	2	47	4.2%
Tumor	51	9	85%

**Table 2.** The correlation between ZNF677 methylation and clinicopathological feature in ccRCC

ZNF677 Methylation Status				
Item	Methylated	Unmethylated	X^2^	*p* value
Age (years)				
≤55	22	4	0.005	0.941
>55	29	5		
Sex				
Male	26	5	0.064	0.800
Female	25	4		
Smoking status				
NO	23	6	1.425	0.232
YES	28	3		
Pathological T stage				
pT1	14	6	5.294	0.021
pT2-T4	37	3		
Lymph node metastasis				
Positive	19	7	5.116	0.023
Negative	32	2		
Distant metastasis				
M0	45	9	1.176	0.278
M1	6	0		
Fuhrman classification				
I–II	21	8	6.974	0.008
III–IV	30	1		
Final Stage				
I	14	6	5.056	0.143
II	17	1		
III	12	2		
IV	8	0

Collectively, these results indicate that, like in other cancers, the low expression of *ZNF677* in ccRCC may be due to its hypermethylation and can be considered as an adverse prognostic factor in ccRCC.

### ZNF677 inhibited 786-0 cell proliferation and induced apoptosis

**Figure 4.**
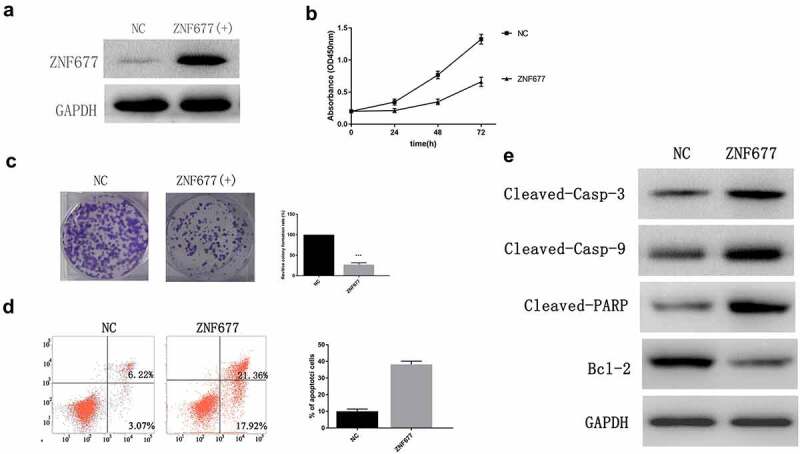
ZNF677 inhibits 786–0 cell proliferation and induces apoptosis. (a) Ectopic expression of ZNF677 inhibited 786–0 cell invasion in vitro evaluated by transwell assay. (b) CCK8 assay showed that ZNF677 suppressed 786–0 cell growth. (c) Clone formation assay showed that overexpression of ZNF677 reduced 786–0 cell colony formation. Colonies larger than 1 mm in diameter were counted: (a) negative control group, (b) ZNF677(+) group. (d) Overexpression of ZNF677 induced 786–0 cell apoptosis. Apoptotic cells of different groups were measured by flow cytometry after 48 h of transduction. The cell populations of Annexin-V+/PI- and Annexin-V+/PI+ were used to assess apoptotic events: (a) negative control group, (b) ZNF677(+) group. (e) Effect of overexpression of ZNF677 on the expression of apoptosis-related genes, measured by Western blot. Cleaved-caspase-3, cleaved-caspase-9 and cleaved-PARP were increased in the ZNF677(+) group cells and not in the control cells, while apoptosis protein BCL-2 was decreased.

To explore the effect of ZNF677 in ccRCC, we established cell lines that stably expressed ZNF677. 786–0 cells were transfected with empty pcDNA3.1 or pcDNA-ZNF677. Western blotting was performed to verify the transfection effect (Figure 4a). CCK8 and colony formation assays were performed to estimate the effect of ZNF677 on cell proliferation. The results of CCK-8 assay showed that cell viability was reduced at 24, 48, and 72 h in ZNF677-expressing cells (Figure 4b), while a reduction of approximately 70–80% in colony formation was observed in ZNF677-expressing cells compared to control cells (Figure 4c). These results demonstrate that ZNF677 inhibited ccRCC 786–0 cell growth.

Flow cytometry analysis was then performed to assess the effect of ZNF677 on cell apoptosis. The results indicated that the percentage of apoptotic cells significantly increased in 786–0 cells after ZNF677 transfection (Figure 4d). On examining the expression of apoptotic protein markers using Western blotting, we found that ZNF677 significantly increased the expression of cleaved-caspase-3, cleaved-caspase-9, and cleaved PARP, and decreased that of BCL-2 (Figure 4e).

### Ectopic expression of ZNF677 suppressed invasion and EMT of 786-0 cells through inactivation of PI3K/AKT signaling pathway

**Figure 5.**
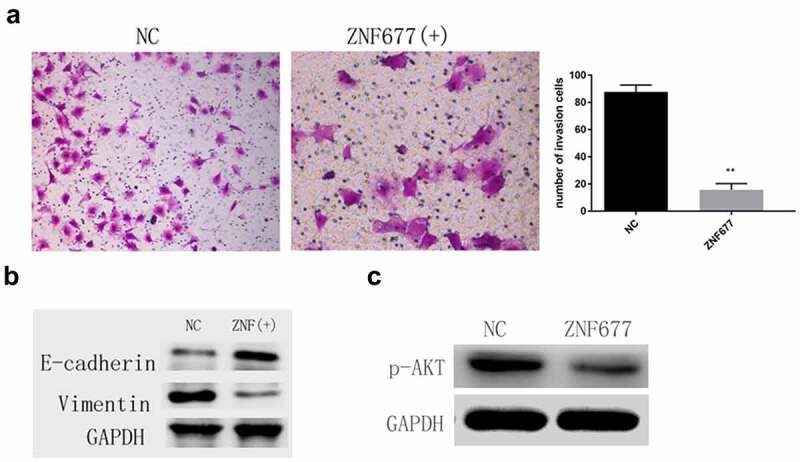
Ectopic expression of ZNF677 suppresses invasion and EMT of 786–0 cells through the inactivation of the PI3K/AKT signaling pathway. (a) Ectopic expression of ZNF677 inhibited 786–0 cell invasion in vitro evaluated by transwell assay. (b) Changes in EMT-related markers between control group cells and ZNF677(+) group cells, measured by Western blot analysis. (c) Changes in p-AKT protein expression between control group cells and ZNF677(+) group cells, measured by Western blot analysis.

Transwell assays were performed to investigate the effect of ZNF677 on 786–0 cell invasion. The results showed that ectopic expression of ZNF677 remarkably decreased the number of invaded cells (Figure 5a). Furthermore, Western blotting indicated that ectopic expression of ZNF677 downregulated and upregulated vimentin and E-cadherin expression, respectively, in 786–0 cells (Figure 5b). These results suggest that ZNF677 inhibits ccRCC invasion by reversing EMT. Previous studies have confirmed that the PI3K/AKT signaling pathway is crucial in ZNF677-mediated invasion and EMT of thyroid cancer cells [14,20]. Thus, we wanted to determine whether AKT signaling is involved in ZNF677-mediated 786–0 cell invasion. Western blot assays revealed that ectopic expression of ZNF677 decreased the expression of p-AKT, suggesting that this molecular pathway may be involved in ZNF677-driven EMT (Figure 5c).

## Discussion

Genetic and epigenetic changes can lead to tumorigenesis. Substantial evidence has verified that epigenetic modifications, such as DNA methylation alterations, nucleosome remodeling, and histone modifications, are involved in the biological process of maintaining normal development and gene expression [21]. Alteration in DNA methylation is one of the most common events and plays an important role in the initiation and development of tumors [22]. It has been recognized that the expression of ZNF family proteins are frequently decreased by DNA methylation in various types of human cancer. For example, ZNF471 is significantly hypermethylated and has low mRNA expression in esophageal cancer compared with that in normal tissues [23]. It has been confirmed that ZNF671 functions as a tumor suppressor in multiple carcinomas via DNA methylation [24,25].

Previous studies have demonstrated that DNA methylation alterations participate in tumorigenesis and are significantly correlated with the clinicopathological features of ccRCC [26]. Dysregulation of gene methylation has been shown to increase ccRCC cell proliferation and invasion and may contribute to the progression and recurrence of ccRCC [26,27]. ZNF677, a member of the ZNF family, has recently been reported to be downregulated in several different types of cancer owing to promoter hypermethylation [13–15]. Combined with data from the TCGA database, the results of the present study demonstrated that *ZNF677* acts as a tumor suppressor gene with significantly higher methylation in ccRCC tumor tissues than that in normal tissues. Clinical data have shown that ZNF677 methylation is significantly and positively associated with clinicopathological features. In addition, hypermethylation was negatively correlated with mRNA expression, suggesting that ZNF677 expression is silenced by aberrant methylation. Moreover, demethylation experiments showed that treatment with Aza and TSA restores the expression of ZNF677 in 786–0 cells. These results indicate that hypermethylation of *ZNF677* causes its downregulation in ccRCC, which is consistent with the results of previous studies on thyroid, lung, and gastric cancers, and implies that *ZNF677* methylation may be a common event in tumorigenesis [13–15]. Furthermore, we used data from the UALCAN platform to explore the potential prognostic value of ZNF677 in ccRCC and found that low ZNF677 expression was associated with poor survival. These data suggest that low expression of ZNF677 is an unfavorable prognostic factor for ccRCC. A recent study reported that ZNF677 is aberrantly methylated in ccRCC and could serve as a noninvasive urine-based diagnostic and follow-up marker for ccRCC [16]. However, the function and mechanisms of action of ZNF677 in ccRCC cells remain elusive. In order to further estimate the potential biological function of ZNF677 in ccRCC, we performed a series of experiments on the proliferation, apoptosis, and invasion of 786–0 cells. The results demonstrated that ectopic expression of ZNF677 significantly inhibited cell proliferation and invasion and induced apoptosis. Our study was the first to investigate the influence of ZNF677 on ccRCC cells malignant biological behavior.Therefore, we inferred that ZNF677 expression might serve as a favorable prognostic biomarker for ccRCC.

Metastasis is the main cause of death in patients with ccRCC. EMT is an essential process in tumor metastasis [28]. Recent studies have proposed that EMT is a key event in the development, invasion, and metastasis of cancers. EMT can promote tumor metastasis and invasion by enhancing the mobility and reducing the adhesion of tumor cells [29]. Decreased expression of epithelial marker genes and increased expression of mesenchymal marker genes are markers of EMT. Therefore, reduced expression of E-cadherin marks the threshold of the EMT process [30]. The influence of ZNF factors on EMT and cancer invasion has been confirmed in various cancers. It has been reported that ZNF703 induces EMT by inhibiting E-cadherin expression, thereby enhancing breast cancer cell invasion and resistance to sorafenib [31]. ZNF281 has been demonstrated to play a crucial role in controlling cellular stemness and EMT by inducing EMT and regulating EMT-associated gene expression in colorectal cancer [32]. ZNF471 suppresses cervical cancer cell invasion by modulating EMT via negative regulation of the Wnt/β-catenin signaling pathway [33]. In this study, we observed that ZNF677 significantly reduced 786–0 cell invasion. Furthermore, ectopic expression of ZNF677 significantly elevated E-cadherin protein expression and reduced vimentin protein expression in 786–0 cells, indicating that ZNF677 suppresses 786–0 cell invasion by reversing EMT. This finding is consistent with those of the studies on thyroid cancer, thus supporting the notion that ZNF677 suppresses cancer metastasis and invasion through the regulation of EMT.

EMT is triggered by many signaling pathways. Aberrant activation of the AKT signaling pathway has been revealed to contribute to cancer cell metastasis and EMT in many cancers [34–36]. Although the precise molecular mechanisms by which ZNF677 is involved in tumor invasion and EMT are currently elusive, activation of the PI3K/AKT signaling pathway has been reported to be involved [15,20]. Siraj et al. demonstrated that overexpression of ZNF677 inhibits thyroid cancer cell invasion, decreases E-cadherin expression, and increases N-cadherin expression. In contrast, knockdown of the PI3K/AKT signaling pathway reduces the expression of N-cadherin, Twist, and Zeb1, accompanied by the expression of E-cadherin, in papillary thyroid carcinoma cell lines [20]. This result indicated that ZNF677 mediates thyroid cancer cell invasion and EMT through the AKT signaling pathway. Therefore, we sought to determine whether the PI3K/AKT signaling pathway is involved in ZNF677-regulated EMT in ccRCC cells. Western blot analysis showed that ectopic expression of ZNF677 significantly elevated E-cadherin protein expression and reduced vimentin protein expression in 786–0 cells, accompanied by the inhibition of cell invasion. The expression of p-AKT protein was also decreased. Thus, the PI3K/AKT signaling pathway may play a role in ZNF677-promoted 786–0 cell EMT. However, additional studies are required to explore the exact molecular mechanisms underlying the effects of ZNF677 on ccRCC cell EMT.

Our study has some limitations. First, no in vivo experiments were conducted and all results were based on public data and cellular experiments; therefore, further validation using in vivo experiments is required. Second, because only the 786–0 cell line was available in our laboratory, only one cell line was examined in the functional experiment. Third, the exact mechanism by which ZNF677 inactivates the AKT signaling pathway remains unclear. Given that ZNF677 is a putative transcription factor, we speculate that it may bind to downstream target genes to regulate the PI3K/AKT signaling pathway. However, the specific mechanisms underlying this effect require further investigation.

## Conclusions

In summary, our findings revealed that ZNF677 is hypermethylated in ccRCC and that ZNF677 inhibits tumor cell proliferation and invasion, and induces apoptosis. Further prognostic analysis indicated that low expression of ZNF677 was associated with shorter OS and that ZNF677 suppresses the invasion and EMT of 786–0 cells through the inactivation of the PI3K/AKT signaling pathway; however, the mechanism underlying this effect remains unknown, and further studies are required to clarify this. This is the first report to evaluate the influence of ZNF677 on ccRCC cells malignant biological behavior. Our results confirm that ZNF677 could be used as a prognostic biomarker for ccRCC.

## Data Availability

All data generated or analyzed during this study are included in this published article.

## Authors’ information

All Authors’ information were listed in the title page.

## Authors’ statement

A preprint has previously been published [37].
